# Evaluating IoT-Based Services to Support Patient Empowerment in Digital Home Hospitalization Services

**DOI:** 10.3390/s23031744

**Published:** 2023-02-03

**Authors:** Patricia Abril-Jiménez, Beatriz Merino-Barbancho, Giuseppe Fico, Juan Carlos Martín Guirado, Cecilia Vera-Muñoz, Irene Mallo, Ivana Lombroni, María Fernanda Cabrera Umpierrez, María Teresa Arredondo Waldmeyer

**Affiliations:** Life Supporting Technologies Research Group, Escuela Técnica Superior de Ingenieros de Telecomunicación (ETSIT), Universidad Politécnica de Madrid, Avda Complutense 30, 28040 Madrid, Spain

**Keywords:** hospitalization at home, IoT, wearables, change management, empowerment, training

## Abstract

Hospitals need to optimize patient care, as, among other factors, life expectancy has increased due to improvements in sanitation, nutrition, and medicines. Hospitalization-at-home (HaH) could increase admission efficiency, moderate costs, and reduce the demand for beds. This study aimed to provide data on the feasibility, acceptability, and effectiveness of the integration of IoT-based technology to support the remote monitoring and follow-up of patients admitted to HaH units, as well as the acceptability of IoT-based solutions in healthcare processes. The need for a reduction in the number of admission days, the percentage of admissions after discharge, and the actions of the emergency services during admission were the most relevant findings of this study. Furthermore, in terms of patient safety and trust perception, 98% of patients preferred this type of digitally-supported hospitalization model and up to 95% were very satisfied. On the professional side, the results showed a reduction in work overload and an increase in trust when the system was adopted.

## 1. Introduction

Life expectancy has increased rapidly, due to improvements in sanitation, nutrition, and medicine. The highest life expectancy in the nineteenth century was lower than that today in all countries of the world, thanks to the sociosanitary advances during the past decades [1]. Hospitals have led these improvements as the main health providers in national health systems (NHS). They are highly complex institutions, offering a wide variety of medical and surgical interventions. However, along with this demographic change, the cost of care continues to increase. In a situation of global economic unpredictability, many health systems are looking for long-term solutions that contribute to the sustainability, accessibility, and appropriateness of health services [1]. In this sense, hospitals, as the main drivers of health care delivery, are required to consider how to optimize patient care.

One of the most extensive practices for increasing admission efficacy, moderating costs, and reducing the demand for beds is hospitalization at home (HaH) interventions. This type of intervention is already common in European hospitals [2,3,4], and they have shown maturity and the generation of health value. HaH could meet the increased demand for healthcare services, while reducing the inconveniences of traditional hospitalization, such as nosocomial infections, pressure sores, lack of privacy, family burden, and the associated cost of hospital beds [5]. Current HaH protocols are made up of a set of well-standardized actions that are tailored according to the patients’ health conditions and socio-economic profile and are provided around daily nurse home visits. These nurses have special training to decide on complementary treatment actions. In addition, physician home visits are scheduled during the admission period, to control the clinical evolution of the patient. 

Although technology is increasingly present in all hospitals, due to the growing use, among others, of IoT devices [6], HaH remains highly dependent on the availability of human resources for its effective adoption. In fact, the need for daily physical visits from highly qualified personnel to monitor, follow-up, and administer treatment to each patient limits the growth of HaH units. Smart technologies can provide pathways and associated services that could guarantee continuous care and safety for more patients with the same human resources [7]. Although telemedicine and remote monitoring solutions based on sensors and other IoT devices are mature enough to be applied in real settings [8], the acceptance of this technology in supporting the daily management of HaH units remains very low [9]. On the contrary, effective technological adoption has allowed chronic disease management to benefit from outpatient procedures in one of the hospital services with the most extensive hospitalization events: remote monitoring technology and digital care solutions allowed these patients to receive regular health checks, health coaching, and general disease monitoring, and, in the long term, prevented acute care episodes [10], reducing associated costs, supporting patient participation, and improving health outcomes [11]. 

In addition, digital care solutions can complement the care of patients with HaH [12]: remote automatic monitoring solutions could provide new data, and continuous monitoring could enable virtual visits while optimizing physical visits, as well as adding professional, technical, and medical support for family members and informal careers. All of this could contribute to reducing the heavy dependence on human resources in HaH units, provide additional operational resources, and potentially favor their expansion. However, there is still a need for evidence of the effective integration of digital technologies into the clinical workflow of HaH, which can support the sustainable and scalable adoption of this technology. Addressing the need for patients and clinicians to use smart technology during admission to HaH can bridge the current gap between participation in technology and clinical procedures. The challenge lies in the need to create trust and systems that ethically and effectively use new digital tools, and maintain the quality of service and patient satisfaction. Unlike with chronic diseases, patients are admitted for 3 to 10 days and require medical and technological expertise for a very short period of time in highly personalized, customized, and specific care routines, while care professionals need to have real-time access to specific monitoring data and alerts, which can prevent any possible risk to the patient. To this end, the transparent usage offered by IoT devices facilitates the skill acquisition of the patients. This study aimed to support the effective definition of new models and approaches that adapt the current ones through the use of available smart digital solutions, which can bring together various expectations and a willingness to ensure the satisfaction of the current and future needs of HaH units. 

The study is part of a project called Better@Home [11], which evaluates the acceptability of adopting a HaH monitoring and follow-up system supported by IoT devices. The objective of this study was to provide evidence on the acceptability and effectiveness of integrating wearable and IoT technologies to support the remote monitoring and follow-up of patients admitted to HaH units, as well as the acceptability of these solutions in care processes for healthcare professionals and patients. Finally, the study aimed to demonstrate the usefulness of IoT technologies in overcoming current HaH barriers resulting from a lack of human resources. The paper is structured as follows: Section 2 describes the proposed technological system and its components, and the methods used to evaluate its feasibility, usability, and other relevant aspects for patient care and professional and patients’ acceptance. Section 3 describes the evaluation results, while in Section 4 and Section 5, the obtained outcomes and their contribution to the objective of the study and conclusions are reported. 

## 2. Materials and Methods

### 2.1. Materials

The Better@Home system for hospitalization at home consists of an integrated automatic remote daily monitoring system to follow patient treatment. The system is an online platform that supports automatic self-monitoring, remote treatment titration, and personalized nutritional recommendations, supervised by a group of healthcare professionals. The main components of the platform are the patient app, which supports personalized training, to support patient acquisition of skills, allowing them to correctly administer and manage their treatment throughout admission, acquire measurements from sensors, and identify incorrect parameters that require professional consultation. Together with the app, patients receive a set of IoT sensors, depending on the requirements of their disease, typically a thermometer, glucometer, weighing scale, pulse oximeter, blood pressure monitor, and electrocardiogram, all with connectivity capabilities and able to support automatic measurement process. The advantage of these connected health devices over conventional ones is that the selected devices can automatically collect the necessary health metrics directly from the patient’s home and send these data to the software application running in the app. The software application algorithms analyze the data and trigger alerts to a professional dashboard in the hospital if needed. All of them are commercially available connected devices with the certification required to be used in a hospital environment, to facilitate practical adoption of the solution. The devices were selected according to a set of communication requirements, such as data that could easily be transmitted using Bluetooth and Wi-Fi protocols. The software application running in the app contains the personalized care pathway for each specific patient. This includes scheduled iterative measurements of patient vital signs. When it is time to measure a vital sign, a reminder is sent to the patient, which it is supported by a automatically launched video that supports the patient in the process of making the specific measurement. Once the measurement is acquired by the sensor, it is automatically sent to the app, typically using Bluetooth or Wi-Fi networks, in a transparent way for the patient. In case a problem is detected during the data transmission, an alert is sent to the professionals, so that they can manage the situation, but the patient can always ask to enter the data manually in case of sensor pairing problems. The devices selected for the study and a summary of the characteristics of the sensors are provided in Table 1.

In addition to the scheduled measures, each patient has a personalized monitoring plan consisting of a questionnaire to control the patient’s progression, which complements the sensors measurements. These were designed based on decision trees established with physicians who evaluate all symptoms, with the aim of having simple and concise questions, while reducing the risk of misinterpretation. To support the patient’s empowerment, part of the gathered information is available in the patient’s apps, to allow them to check the evolution of their symptoms. 

Another component of the platform is the clinical management system, which allows physicians to remotely monitor the status of patients, using real time data from the sensor in the patient’s home. Data gathered by the sensors are displayed in a web visualization dashboard in the hospital. A color-based triage system (red-yellow-green) is used to automatically categorize the patients according to their health risk and support the monitoring of their progress. In addition to triage and alerts, the dashboard provides evolution graphs and other outcomes, so that the clinical team can implement the necessary procedures, according to the hospital’s protocol of action. In addition, a special type of alert is automatically launched in case of an abnormal patient situation or if a patient has requested urgent assistance. Finally, the system allows for the generation of configurable and customizable reports and structured data extraction. 

**Figure 1:**
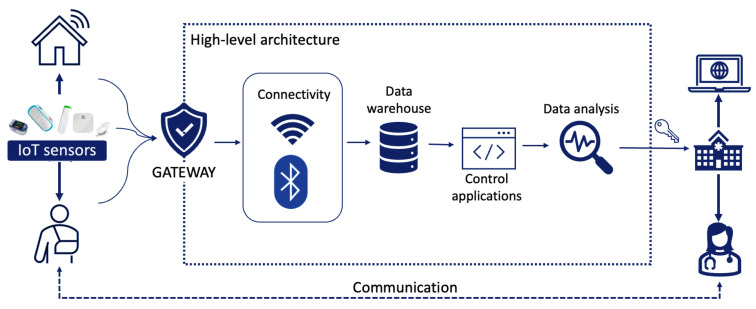
Process and IoT devices flowchart.

A virtual communication channel was included in the professional platform, to allow the healthcare team to make video calls with the patient. This allows controlling the evolution of patients, without the need to go to the patients’ homes in case additional information is needed. It is the hospital professionals who establish the day and time of the video call. When the patient first accesses the system, he or she will remain in the “waiting room” until the doctor allows access. The high-level architecture of the proposed system is provided in Figure 1.

### 2.2. Methods

The evaluation of the proposed digital HaH system was carried out following a multidomain approach, aimed at evaluating the technical feasibility of the solution, the efficiency of the system with respect to the current HaH practices, and the empowerment of the patient, to improve self-care and self-management of the disease. Finally, the acceptance of the system by professionals was also measured, to assess the resistance to change and the workload of the involved health personnel when introducing these digital solutions. 

The feasibility and efficiency evaluation of the system was performed using a key performance indicator (KPI) definition approach based on common indicators in health economics for the same type of hospital services. To select KPIs, we conducted a literature review [13,14]; the ones used in the present study are presented in Table 2. Their values were collected within the logs and internal statistics of the HaH unit involved in the study, and before and after the study.

**Figure 2:**
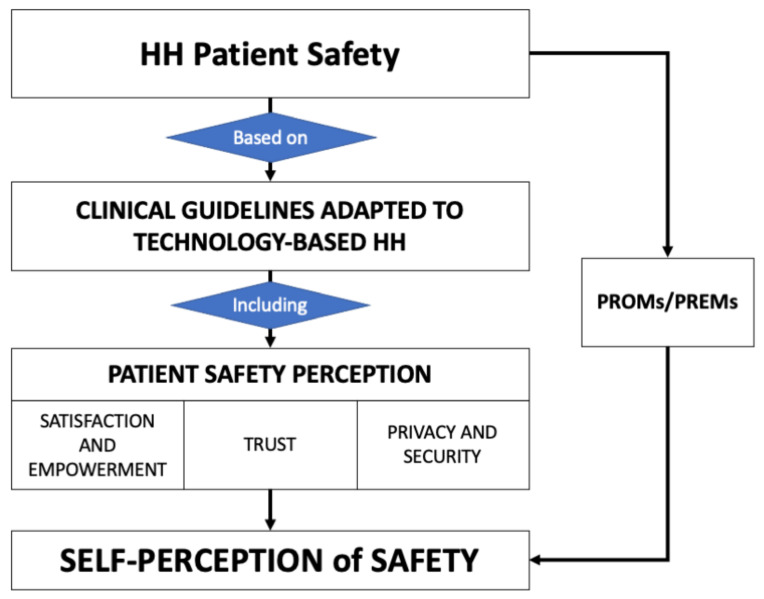
Conceptual framework to generate the Better@Home assessment questionnaire.

In addition to the feasibility and efficiency assessment, the impact on patients and professionals involved was also evaluated. There is no consensus on the best instrument to measure patient skill acquisition and the empowerment of the patient, this is because the selection of one or the other strongly depends on the situation in which the instrument will be used [15,16]. To overcome this problem, the research team developed a short self-administered questionnaire, to collect specific items associated with satisfaction, acceptability, patient experience, and empowerment. This questionnaire was based on detailed research on the current instruments used to measure the outcomes of patient experience and its relation with their cultural background [17]. After conducting a literature review, a total of 10 instruments were selected. For instance, IEXPAC [18] evaluates the chronic patient experience in terms of the use of technological solutions, educational empowerment, and self-management, which is useful to evaluate the acquisition of skills of admitted patients and the trust of selected devices; PES-Q [16] evaluates patients’ empowerment and acceptance of the solution; SUS [19] is traditionally used to evaluate the usability of digital systems. These were examined with 10 professionals during a discussion session. The results were analyzed and organized within a conceptual framework composed of different domains that make up the perception of HaH safety (Figure 2). 

At the end of this process, a list of 35 questions emerged that describe key factors that contribute to patient satisfaction, comfort, acceptability, and empowerment. In the second stage, a discussion group was formed with 10 HaH experts, composed of five physicians and nurses, three IT health developers, two patient-engagement experts, to discuss the elements needed to overcome barriers to the acceptance of HaH for patients, the burden of treatment, the existing measures and evaluation frameworks, and the most relevant domains to include in the questionnaire. The resulting questionnaire consisted of 19 common questions and 20 that depended on the type of disease, in Spanish and based on a seven-point Likert scale, between strongly agree (7) and strongly disagree (1) in four domains (Table 3): (1) patient understanding of their role, since the patient has an active role in the provision of care during administration; (2) acquisition of health knowledge of patients, which measures the level of medical skills and education acquired during admission, which has a direct effect on the quality of care; (3) technical, functional, and digital knowledge of patients, which evaluates the usability, satisfaction, and trust perception of the proposed digital system; and (4) patient empowerment, which evaluates four domains directly related to patient quality of care and treatment adoption [20,21]. The questionnaire is currently undergoing a validation process. 

In the case of professionals, the objective was to assess the knowledge, skills, outcomes, expectations, self-efficacy, motivation, and trust in the adoption of a new digital solution. Three validated self-administered questionnaires were used. The MBI-HSS (Maslach Burnout Inventory; Human Services Survey) [22], used successfully in medical settings to assess the impact of staff when making changes to workflow and selected to obtain valuable information on professional working conditions and the impact of including new workflows and tools in their daily routine; the MBI-HSS consists of a 15-item questionnaire divided into three subscales, including emotional exhaustion (5 items), depersonalization (4 items), and personal achievement (6 items), scored from 1 to 5. For emotional exhaustion and depersonalization, higher scores mean more severe job burnout, while lower scores in personal achievement mean more severe job burnout. This instrument was administered at baseline, to understand the professional context before using the new solution and at the end of the study. The PSSUQ (Post-Study System Usability Questionnaire) version 3 [23] was used to evaluate the usability and usefulness of the professional tools. It is a 16-element questionnaire, scored with a seven-point Likert scale (+NA option) between strongly agree (7) and strongly disagree (1). The CREAC questionnaire measures trust building capability. In particular, this instrument aims to measure the trust and confidence of the professional in the system, with respect to the training they received to use the new digital solution, providing valuable information about the trust and confidence of the professionals in the new solution. This questionnaire analyzes how the professional acquires the skills to use the system and contributes to building trust in the patient. It has 12 items, scored with a seven-point Likert scale (+NA option) between strongly agree (7) and strongly disagree (1).

These questionnaires were completed with a sociodemographic questionnaire used to identify the different social and working conditions of the involved professionals. The different domains measured in the professional evaluation and the instruments used to evaluate them are presented in Table 4. 

## 3. Results

From February 2021 to December 2021, a total of 208 patients participated in the study. The sociodemographic characteristics of each of the current samples in the group are shown in Table 5 (patients) and Table 6 (professionals). Most of the patients evaluated the HaH with infections (both urinary and tissues) and in the case of lung diseases, COVID-19 was one of the most frequent causes of admission (n = 57, 87.30%). 

### 3.1. KPIs Results

The KPI outcomes obtained after evaluating the results in each patient showed a significant improvement in the hospitalization service compared to the traditional service. It is especially relevant in reducing face-to-face intervention, which has the potential to increase the number of beds without increasing the number of medical personnel. The reduction in readmissions was not so high, but this was significant because of the implications for the disease had for self-management, which reduces the number of exacerbations in short- and medium-term, see Table 7.

### 3.2. Main Outcomes for Patients 

Of the patients admitted to the emergency room as eligible for an HaH intervention, 90.4% accepted to participate in the program (approximately 60% of the total of patients admitted to the emergency room during the period with the same type of disease and admission cause). Of these, 75.96% completed the evaluation. In general, the HaH intervention was rated as positive in the four evaluated domains, with an agreement rate of more than 6, ranging from 5 to 7 per domain (Table 8). The domain with the highest rating was the acquisition of health literacy from patients, and the lowest rated was the empowerment of patients. 

In addition to these data, the mode of admission days changed from 11 days in traditional HaH to 6 days, implying a 54% reduction, while the mode of required professional visits changed from 3 to 1. During the study period, no adverse events were documented, and 18 patients (9.23%) required emergency assistance. No statistically significant differences were found between the responses to the questionnaire and the sex, age, or disease (*p* > 0.05) of the patients.

Finally, 98% of the patients considered that if they had to be readmitted, they would like to use digital HaH again, because they could integrate telemedicine and IoT devices into their lives without problems and felt confident, obtaining a high satisfaction rate from over 95% of the patients. 

Thematic analysis of free-text comments led to the identification of the process and impact as the two main themes: identified barriers and improvement suggestions and the willingness to use this mode of hospitalization in the future. Most of the respondents reported very positive experiences, while few patients provided information on difficulties due to not being physically attended by a healthcare professional or barriers when using the digital solutions. The feedback was most encouraging about the benefits of being able to stay at home and being proactive with their daily care plans. 

The percentage of patients who used each type of IoT device available is shown in Table 9.

### 3.3. Main Results for Professionals 

The results of the baseline and final evaluation of the professional intervention program performed are shown in Table 10 for the MBI-HSS and CREAC questionnaires.

The analysis of professional comments suggested that the main barriers identified were related to the need to educate and inform all users. The technical strategies adopted with the inclusion of monitoring technologies were considered very positive and were not considered a barrier, rather a facilitator to improving their effectiveness.

## 4. Discussion

The presented study provides prerequisites for the adoption of a digital solution to support HaH units, offering the opportunity to evaluate how remote monitoring systems and telemedicine can influence the quality of life of patients admitted to a HaH service, but also their health empowerment and satisfaction at discharge, as well as providing initial evidence of their feasibility of implementation and integration into national health systems from a technical point of view. Furthermore, the study aimed to provide evidence on the impact of introducing these remote monitoring and support tools into a HaH unit workflow, which is key to the implementation of the final service [24]. By using already mature technologies with proven effectiveness in reducing service costs and maintaining service quality [25], there is the opportunity to extend the benefits of HaH, increasing the number of beds available in current units. Recent studies have shown the benefits of HaH, but also its barriers and future challenges [12,26]. Therefore, they are many points in common with other experiences and the remote collection of health records already evaluated using different methods and tools, and in different research areas.

The intervention involved more than 90.4% of hospital patients admitted to the emergency department. The number of adverse events was lower than the hospital average; only 9% of the patients needed urgent intervention, even considering that part of the study was carried out under the adverse conditions of the COVID-19 pandemic, with a great reduction of emergency services in the same period [23,24,25]. This also highlights the feasibility and adaptability of the solution from a technical point of view, since it allowed for fast integration and management of the monitored patients in such a complicated situation. Four disease protocols, with up to 16 different pathways, were successfully implemented with the selected set of IoT devices, which demonstrate the computational efficiency of the proposed solution. 

Although there were difficult conditions when deploying the system, the average number of hospitalization days was six, while in traditional HaH this was 11; the number of required visits was reduced from three to one on average [27]. This is also good evidence of the usefulness of the proposed real-time transmission of data and alert management, generating a situation of trust and reliability regarding the proposed solution and the associated devices. 

The influence of the pandemic on the results of professionals was also observed. They were also very positive, in general, but with signs of overwork and burnout due to stressful situations. This was also highlighted in other research studies aimed at assessing anxiety and burnout levels in emergency medical workers during the COVID-19 pandemic; however, our findings showed better results at the end of the evaluation, with lower burnout rates in the three domains [28,29]. However, some positive effects were observed at this point in the study. The proposed solution helped to create a secure environment, where professionals do not feel overwhelmed by the new processes and tools, even during the problematic situation they were facing, according to the improvements in the CREAC and MBI-HSS results [30]. These results show professionals feel confident in the solution, which helps build trust with patients. 

Both groups of participants were positive about the usefulness and effectiveness of the intervention. Taking into account the heterogeneity of the medical, social, educational, educational, and demographic conditions of the patients, the system demonstrated personalization, adaptability, and feasibility. 

In the case of patients, the worst rated dimension was patient empowerment. Taking into account that this was the first time they were hospitalized at home with this technical support, we consider that the lower rates of self-perceived empowerment may have been due to the subjective need of depending on healthcare professionals [31]. Further analysis should focus on analyzing the empowerment of patients who were previously hospitalized at home. 

Our findings demonstrate that the integration of different methodological approaches from various research fields opens an opportunity to bridge the existing lack of evidence on the applicability of digital technologies. Interventions have been previously developed to promote digital education in home care and specifically in HaH [32,33]. Most of them did not target a wide spectrum of ages and socio-economic profiles, nor address the challenge of building the educational capacity of patients and professionals [34,35]. When the target group is homogeneous, the educational content is more specific and well-defined. Conversely, addressing different target groups entails difficulties in delivering and adapting the educational content and information [31,32,34]. One of the challenges of developing this intervention was in allowing professionals and patients to learn how to use multiple devices and services in a few hours, without compromising patient safety and avoiding any type of risk. Based on studies published previously [36,37,38], we obtained the background necessary to describe the problem, define the intervention, and plan data collection.

The evaluation results demonstrated high levels of satisfaction, both in patients and professionals. This is highly relevant in the case of patients, since their perceived service quality was reinforced by their trust in the solution, the easy communication with the professionals, and the comfort of being admitted in a familiar environment, as it was their own home. 

There are some limitations to our study. A larger sample size would be necessary to improve the statistical power of the outcomes. Some of the results of the questions did not reach significance as a result of the sample size. In addition, some of the questionnaires used for the evaluation were related to subjective feelings and could result users to multiple interpretations, turning them into a source of bias. This risk is currently being reduced, as the questionnaire is in the validation process. Furthermore, this study focused on aspects of human–human interaction acceptance; more studies will be conducted in the future, in more hospital settings, on the reliability and stability of the proposed system. Future evaluations will help identify whether the intervention effectively contributes to improving patient-reported outcomes and reduces the workload and costs associated with health professionals, as well as supporting patients to improve their health outcomes and contributing to the efficient and effective adoption of digital HaH programs. 

## 5. Conclusions

A HaH system such as the one proposed in this study facilitates the adoption of HaH protocols, without the need for training and the involvement of more human resources. The continuous monitoring system facilitates the continuous follow-up of the patient’s status from their home, without the need for daily nurse visits, with very good results in patient outcomes, satisfaction, and trust. Professionals also benefited from higher ratings and improvements in their daily management of the process factors, such as a reduction of burnout feelings. 

## Figures and Tables

**Table 1:** Devices based on the IoT.

IoT Device	Service Offered	Device and Commercial Description
Thermometer	Ear infrared thermometer, fast reaction time (2 s, IrDA communication with 2300	D-1261 from the Prior Medical System (VB Den Haad, Holland)
Glucometer	A glucometer is a home measurement system that the patient can use to test the amount of glucose in the blood.	In this case, any commercial glucometer could be used, as the value is manually introduced in the patient’s application
Weighing Scale	A body scale to facilitate weight control. It is an automatic scale that does not need to be turned on. Bluetooth^®^ communication	UC-351PBT-Ci from AND (Oxfordshire, United Kingdom)
Pulsioximeter	Small, lightweight, and portable wireless pulse oximeter. Code lights allow the patient to know if the pulse oximeter is placed correctly. Bluetooth^®^ communication	Nonin3230 (Amsterdam, Netherlands)
Blood-Pressure Monitor	Arm sphygmomanometer designed to accurately measure blood pressure. Bluetooth^®^ communication	AND UA-767 PlusBT-Ci (Oxfordshire, United Kingdom)
ECG	Patches are only for single use and obtain the signal from the electrocardiogram to evaluate rhythm and cardiac function by recording the electrical activity of the heart.	

**Table 2:** Definition of KPIs.

KPI	Explanation
Percentage decrease in face-to-face intervention	Percentage decrease in physician and nurse visits for face-to-face intervention at patients’ homes. A decrease in this rate is expected, as these visits will be complemented by remote follow-up.
Percentage increase in remote intervention rate	Percentage increase in remote interventions in hospitalized patients at home. An increase in this rate is expected, as these visits will increase due to the implementation of remote monitoring.
Percentage decrease in readmissions 72 h after hospitalization at home discharge	Readmissions 72 h: Considers readmission as admission 72 h after HaH discharge
Percentage decrease in readmissions 30 days after hospitalization at home discharge	Readmissions 30 days: Considers readmission as admission 30 days after HaH discharge
Percentage decrease in readmissions to hospital for conventional admissions (CH) from hospitalization at home.	Readmissions to CH: Considers readmission as admission to the CH of HaH.

**Table 3:** Survey measures completed by patients.

Domain	Objective	Items
**Patient understanding of their role**	Evaluate communication between patients and professionals about diagnoses and names, purpose and adverse effects of treatment, patient understanding of the treatment plan during hospitalization, and level of health knowledge before admission.	3 items
**Acquisition of health literacy in patients**		
Education and capacity building	Evaluates the perception of an effective intervention	3 items
Knowledge and skills acquired by the patient in health	Evaluates effective skill acquisition for treatment management	4 items
**Technical, functional, and even digital literacy for patients**		
Trust and Security	Represent how patients depend on digital devices and treatment	3 items
Satisfaction	How patients are happy with digitalized hospitalization services	3 items
Usability	How easy are the devices and digital systems to use	20 items
**Patient empowerment**	How patients can deal with their health conditions during hospitalization and expect to continue managing their health conditions after discharge	3 items

**Table 4:** Survey measures completed by health professionals.

Domain	Measures	Administrated at
Work environment and skill acquisition	PSSUQ	End of the study
Trust and confidence in the solution	CREAC	End of the study
Viability and operability of new work paths	MBI-HSS	Baseline and end of the study
Sociodemographic and working variables	Survey	Baseline

**Table 5:** Sociodemographic characteristics of patients, including number of days in the HaH unit and number of visits by health professionals.

Patients	N = 208
Gender	
Male	110
Female	84
Unknown	15
Age	62.42 (SD = 23.76)
Disease	
Pulmonary disease	63
Tissue infections	56
Urinary infections	78
Cardiovascular disease	11
Number of days	6
Number of visits	1

**Table 6:** Sociodemographic characteristics of professionals.

Professionals	N = 40
Age	37.75
Gender	Male (n = 9). Female (n = 29) Unknown (n = 2)
HaH experience (year)	4.1
Working	Full-time (n = 38) Shift basis (n = 2)

**Table 7:** Results of the evaluation of KPIs for feasibility.

KPI	Result
Percentage decrease in face-to-face intervention	24% (hospital medical doctors) + 14% (nurses)
Percentage increase in the remote intervention rate	18% decrease in phone calls + 100% increase in videoconferences
Percentage decrease in readmissions 72 h after hospitalization at home (HaH) discharge	2%
Percentage decrease in readmissions 30 days after hospitalization at home (HaH) discharge	5%
Percentage decrease in readmissions to hospital for conventional admissions (CH) from hospitalization at home (HaH).	2%

**Table 8:** Average results for the four main domain questions in the patient survey.

Domain	Mean
Understanding of the patient about their role	6.23 ± 0.23
Acquisition of health knowledge by patients	6.86 ± 0.48
Technical, functional, and digital literacy of patients	6.17 ± 0.81
Patient empowerment	5.62 ± 1.12

**Table 9:** Average Results of Using IoT Devices.

Device	Number of Participants Using the Device	Percentage of Satisfaction with the IoT Device
Thermometer	89%	92%
Glucometer	67.12%	79.77%
Weighing Scale	3%	57%
Pulse oximeter	99%	94%
Blood-Pressure monitor	100%	87%
ECG	16.5%	95%

**Table 10:** Average results of the professional survey intermediate evaluation.

Domain	Mean ± SD (Baseline)	Mean ± SD (Final)
**MBI-HSS**		
Emotional exhaustion	3.12 ± 0.56	3.23 ± 0.32
Depersonalization	2.12 ± 0.82	2.64 ± 0.56
Personal accomplishments	3.24 ± 0.85	3.22 ± 0.82
**CREAC**		
Trust-building capacity	3.25 ± 1.10	4.18 ± 1.25

## Data Availability

The data sets generated and/or analyzed during the current study are not publicly available, due to hospital restrictions, but are available from the corresponding author upon reasonable request.
